# Sex differences in microRNA-mRNA networks: examination of novel epigenetic programming mechanisms in the sexually dimorphic neonatal hypothalamus

**DOI:** 10.1186/s13293-017-0149-3

**Published:** 2017-08-15

**Authors:** Christopher P. Morgan, Tracy L. Bale

**Affiliations:** Department of Biomedical Sciences, University of Pennsylvania School of Veterinary Medicine, 380 South University Ave, 410F Hill Pavilion, Philadelphia, PA 19104 USA

**Keywords:** microRNA, Argonaute HITS-CLIP, Sexual differentiation, miRNA-target gene networks, Epigenetic programming, Hypothalamus

## Abstract

**Background:**

Sexual differentiation of the male brain, and specifically the stress circuitry in the hypothalamus, is primarily driven by estrogen exposure during the perinatal period. Surprisingly, this single hormone promotes diverse programs of sex-specific development that vary widely between different cell types and across the developing male brain. The complexity of this phenomenon suggests that additional layers of gene regulation, including microRNAs (miRNAs), must act downstream of estrogen to mediate this specificity.

**Methods:**

To identify noncanonical mediators of estrogen-dependent sex-specific neural development, we assayed the miRNA complement of the mouse PN2 hypothalamus by microarray following an injection of vehicle or the aromatase inhibitor, formestane. Initially, multivariate analyses were used to test the influence of sex and experimental group on the miRNA environment as a whole. Then, we utilized traditional hypothesis testing to identify individual miRNA with significantly sex-biased expression. Finally, we performed a transcriptome-wide mapping of Argonaute footprints by high-throughput sequencing of RNA isolated by cross-linking immunoprecipitation (Ago HITS-CLIP) to empirically characterize targeting relationship between estrogen-responsive miRNAs and their messenger RNA (mRNA) targets.

**Results:**

In this study, we demonstrated that the neonatal hypothalamic miRNA environment has robust sex differences and is dynamically responsive to estrogen. Analyses identified 162 individual miRNAs with sex-biased expression, 92 of which were estrogen-responsive. Examining the genomic distribution of these miRNAs, we found three miRNA clusters encoded within a 175-kb region of chromosome 12 that appears to be co-regulated by estrogen, likely acting broadly to alter the epigenetic programming of this locus. Ago HITS-CLIP analysis uncovered novel miRNA-target interactions within prototypical mediators of estrogen-driven sexual differentiation of the brain, including Esr1 and Cyp19a1. Finally, using Gene Ontology annotations and empirically identified miRNA-mRNA connections, we identified a gene network regulated by estrogen-responsive miRNAs that converge on biological processes relevant to sexual differentiation of the brain.

**Conclusions:**

Sexual differentiation of the perinatal brain, and that of stress circuitry in the hypothalamus specifically, seems to be particularly susceptible to environmental programming effects. Integrating miRNA into our conceptualization of factors, directing differentiation of this circuitry could be an informative next step in efforts to understand the complexities behind these processes.

**Electronic supplementary material:**

The online version of this article (doi:10.1186/s13293-017-0149-3) contains supplementary material, which is available to authorized users.

## Background

Biological sex is a strong predictor of many aspects of neurodevelopmental disorders, including incidence, presentation, and therapeutic outcomes [1, 2]. Endophenotypes of neuropsychiatric disease, such as increased stress responsivity, display sex-biases across the normal population [1]. Indeed, it has been suggested that the disrupted development of these normal sex differences may contribute to the etiology of disease [3]. While the neural basis for most individual traits is incompletely understood, sex differences in the brain have consistently been identified at all levels of neurophysiology [4].

Much of our understanding of the mechanisms responsible for establishing sex differences derives from the organizational/activational hypothesis of sexual differentiation [5]. According to this hypothesis, early-life exposure to gonadal hormones, during specific windows of sensitivity, directs sex-specific developmental processes. This organized neurocircuitry is then activated by the adult steroid hormone environment to express sex-appropriate behavior and physiology [6–8]. This framework, though originally established in studies of rodent reproductive behavior, has been extended to other sex-biased traits, including stress responsivity [9, 10]. In males, the brain is organized by a rise of testosterone during the perinatal-sensitive period. This testosterone is converted to estrogen by a neuronal aromatase in appropriate cell populations, where it alters gene expression to masculinize and defeminize key neurocircuitry [5, 6]. While the primary effector, estrogen, is shared, the cellular processes necessary for appropriate development vary widely between sexually divergent traits and brain regions. This emphasizes the necessity of additional downstream sex-biased epigenetic factors, such as microRNAs (miRNAs), to ensure the expression of appropriate gene networks [11, 12].

miRNAs are small non-coding RNAs required for the normal development of all tissues [13–15]. While novel noncanonical functions have been identified, miRNAs act primarily as part of the Argonaute-containing RNA-induced silencing complex (RISC complex) to regulate post-transcriptional gene expression. Mature miRNAs guide the RISC complex to specific mRNA targets, identified by regions of sequence homology, miRNA recognition elements, often present in the target’s 3′ UTR. Argonaute proteins act at the interface between miRNAs and their target mRNA to mediate the functional consequences of these interactions, typically resulting in destabilization and subsequent degradation of the transcript [16–18]. The majority of mRNAs are targeted by one or more miRNAs, and similar to transcription factors, a single miRNA may regulate the expression of hundreds of different genes [19–21]. Together, these properties suggest that miRNAs are major components of an integrated gene expression regulatory mechanism and may be poised to dynamically program sex differences in neurodevelopment [22].

We have previously shown that the neonatal brain miRNA environment is sexually dimorphic and dynamically responsive to organizational hormones [23]. Therefore, in these studies, we focused specifically on the neonatal hypothalamus, a brain region involved in the expression of sex differences in neuroendocrine processes, including growth, stress, metabolism, sleep, circadian rhythm, reproduction, and feeding. We first compared patterns and identified miRNAs with sex-biased expression. Then, to determine if the dramatic sex differences in these miRNAs were driven by estrogen vs. sex chromosomal regulation, we manipulated the availability of neuronal estrogen using treatment with the aromatase inhibitor, formestane. Finally, utilizing Argonaute (Ago) HITS-CLIP (high-throughput sequencing of RNAs isolated by crosslinking immunoprecipitation), we identified that the mRNA targets of these miRNAs actually co-localized within the RISC complex [24]. This method allows us to move beyond more traditional bioinformatics-based approaches by refining candidate sequence-based miRNA-binding sites to those that are bound by Ago, and are therefore likely functional [25]. Ago HITS-CLIP is currently one of the most effective ways to experimentally validate miRNA-targeting relationships. This is particularly true for identifying these connections at the omics-level in tissues. When combined, these data should shine light on the cellular processes necessary for appropriate neuronal sexual differentiation, which may be vulnerable to disruption by insults or susceptible to therapeutic manipulations.

## Methods

### Animals

Male C57BL/6J and female 129S1/SvImJ mice were obtained from Jackson Laboratories and subsequently used as breeding stock to produce C57BL/6J:129S1/SvImJ hybrids (F1 hybrids). The hybrid vigor of this background strain provides a reproducible balance of stress responsivity, behavioral performance, and maternal care used reliably in our mouse models [23]. All mice were housed in a 12-h light/dark cycle with ambient temperature 22 °C and relative humidity of 42%. Food (Purina Rodent Chow; 28.1% protein, 59.8% carbohydrate, 12.1% fat) and water were provided ad libitum. All studies were performed according to experimental protocols approved by the University of Pennsylvania Institutional Animal Care and Use Committee, and all procedures were conducted in accordance with the NIH Guide for the Care and Use of Laboratory.

### Formestane administration

Pups were treated with the aromatase inhibitor, formestane, or vehicle on the morning following parturition. To control for litter effects, within litters, male pups were randomly assigned to receive 20 μg of formestane (Sigma-Aldrich) in 20 μl sesame oil with 10% ethanol or vehicle injections. This dose, after adapting for differences in rat versus mouse neonatal weight, was previously shown to reduce male hypothalamic estrogen to levels found in normal females at this age [26]. All female pups received vehicle injections. Injections were administered subcutaneously between the shoulders, and the injection site was treated with New Skin liquid bandage to prevent leakage.

### Dissection of the PN2 hypothalamus

Pups were sacrificed 24 h after treatment, post-natal day 2 (PN2). Whole brains were dissected, and placed in a neonatal mouse brain slicer matrix (Zivic Instruments), which was kept on ice. A 2-mm coronal slice was collected from approximately 2.5–4.5 mm posterior of the anterior edge of the olfactory bulb according to the Atlas of the Developing Mouse Brain (PN1) [27]. This slice was placed on its anterior surface and the whole hypothalamus was grossly dissected with a scalpel. For miRNA microarray analysis, the hypothalamus was frozen in liquid nitrogen and stored at −80 °C prior to assay. For Ago HITS-CLIP, the dissected hypothalamus was immediately placed on ice in HBSS for immediate processing.

### PN2 hypothalamus miRNA microarray

Total RNA was extracted from the PN2 hypothalamus using TRIzol reagent (Invitrogen), and total RNA was isolated according to the manufacturer’s protocol. One microgram each from two littermates was pooled (pooled samples: F/Veh *n* = 5, M/Form *n* = 7, F/Veh *n* = 6) and submitted for microarray analysis (Affymetrix GeneChip miRNA 3.0 Array). Microarray analyses were performed as described previously with minor modifications [28]. R (version 2.14.2) was used with the limma package to generate gene expression values and to fit linear models with the predictor value of sex to these data [29, 30]. Based on the observation that in microarray experiments unexpressed transcripts can be most reliably detected by their low variability across all samples, miRNAs with total variance values in the lowest 0.25 quantile were excluded [30]. Thresholds for multiple comparisons in determining sex-biased miRNAs were set at a false discovery rate (FDR) ≤ 0.05. Multivariate analyses of miRNA expression were performed using SIMCA-P software (UMETRICS).

### Argonaute high-throughput sequencing of RNA isolated by cross-linking immunoprecipitation (Ago HITS-CLIP)

At the time of tissue harvest, the dissected PN2 hypothalamus from a single pup was placed in 8 mL of cold HBSS and lightly broken up with repeated pipetting. This suspension was placed in a 10-cm tissue culture plate, kept on ice, and UV-irradiated to covalently link RNA-protein complexes (3 × 400 mJ/cm^2^ in a Stratalinker). After crosslinking, the tissue suspension was pelleted and frozen in liquid nitrogen until subsequent immunoprecipitation. For immunoprecipitation, cross-linked tissue pellets from five pups, each from a different litter were pooled (resulting in: F/Veh *n* = 2, M/Form *n* = 2, M/Veh *n* = 2). When pooled, each biological replicate was derived from approximately 25 mg of total hypothalamic tissue from five animals. HITS-CLIP was performed using the monoclonal anti-Ago antibody 2AE (courtesy of the Mourelatos Lab) as previously described [24, 31]. Libraries were generated for RNA-seq (consisting of both mRNA and miRNA fractions) and sequenced using Illumina chemistry.

Libraries were processed as previously described [32]. Because we started with a limited quantity of tissue, after sequencing, the library reads from the two biological replicates per treatment group were combined to maximize read counts. This generated a single set of sequencing data for each treatment group (F/Veh, M/Form, and M/Veh). Reads were then processed to trim adapter sequences. Sequences were aligned to miRbase, for miRNAs, or RefSeq mRNAs for targets. Ago footprints were identified by coalescing aligned reads into clusters. For each position in RefSeq annotated mRNAs, we calculated the total number of reads with an alignment that started at that position. We then arranged all locations in order from most aligned reads to the least. We processed each position in this order. A position was counted as a unique footprint if it was at least 10 bp away from an existing footprint; otherwise, it was combined with the adjacent pre-existing footprint. The final start position of a footprint was defined as the first nucleotide that was used to create the footprint. The strength of a footprint is the total count of all reads assigned to a footprint. These footprint counts were then normalized in two steps: (1) they were converted to read fractions and then (2) they underwent quantile normalization between samples, resulting in a final footscore [reads per million (RPM)] for each Ago footprint. Finally, miRNA-targeting relationships were predicted using miRanda on all RefSeqs mRNAs. A miRNA-mRNA connection was initially called if the miRNA was present in the Ago-short library at a minimum of 100 RPM and was predicted to bind to a RefSeq mRNA that fell within 50-bp downstream of the start of an Ago footprint. This connection between a miRNA and Ago footprint constituted a miRNA regulatory element.

### Databases/software

miRNA annotations were derived from miRBase v.21 [33]. miRNA cluster assignments were obtained from MetaMirCluster [34]. Transcription start site (TSS) mapping data was produced by the FANTOM5 Consortium and obtained as a UCSC Genome Browser public track (FANTOM5 TSS peaks [robust]) [35]. Ago footprint annotations and alignment were derived from RefSeq mRNA [36]. Genomic coordinates reference *Mus musculus* genome assembly MGSCv37 (mm9) [37]. The network of Ago HITS-CLIP connections was generated using Cytoscape v.3.1.1 [38]. Clustering of enriched Gene Ontology terms (GO Biological Processes release 03/20/2014) was performed with the Cytoscape plug-in ClueGo v.2.1.6 [39, 40]. Previously validated miRNA-target interactions were accessed through the database DIANA-Tarbase v7.0 [41].

## Results

### Sex differences in the miRNA environment of the neonatal hypothalamus

**Fig. 1:**
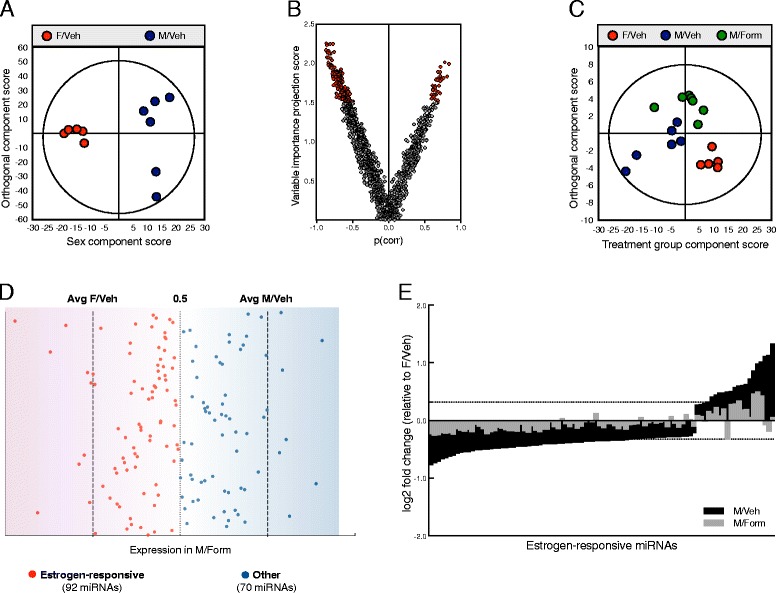
The miRNA environment of the neonatal (PN2) hypothalamus is sexually dimorphic and dynamically responsive to estrogen. **a** OPLS-DA score plot of a model generated from the expression of 1407 miRNAs in the PN2 hypothalamus shows clear separation between control female (F/Veh) and male (M/Veh) groups along the predictive component (sex) (*x*-axis: *R* ^2^ = 0.45, *Q* ^2^ = 0.43, p[CV-ANOVA] = 0.04). **b** Volcano plot based on this multivariate model demonstrates that a clear majority of miRNAs was downregulated in M/Veh relative to F/Veh. F/Veh, (*n* = 5); M/Veh, (*n* = 6).**c** OPLS-DA score plot of a model generated from the expression of 162 miRNAs with a significant sex-bias (FDR ≤ 0.05) in the PN2 hypothalamus following a single PN1 injection of the aromatase inhibitor, formestane (20 μg) (Form), or vehicle (Veh) shows clear separation between F/Veh, control male M/Veh, and M/Form groups along the predictive component (treatment group) (*x*-axis: *R* ^2^ = 0.57, *Q* ^2^ = 0.39, p[CV-ANOVA] = 0.06). The F/Veh group is clustered around one central component, while the M/Form group is a distinct intermediary between F/Veh and M/Veh groups. **d** A plot of the mean M/Form expression of individual sex-biased miRNAs (*y*-axis) along a continuum between mean F/Veh and M/Veh expression (*x*-axis) [M/Form expression on continuum = (M/Form−F/Veh)/(M/Veh−F/Veh)]. While the magnitude of the basal sex difference varied between miRNAs, M/Form expression of 92 of these miRNAs was closer to F/Veh than M/Veh, suggesting that the sex-biased expression of these miRNAs is dependent on estrogen (*red dots*). **e** Seventy-one of these estrogen-responsive miRNAs were reduced in the M/Veh group relative to F/Veh. This suggests they are downregulated by estrogen. F/Veh, (*n* = 5); M/Veh, (*n* = 6), M/Form, (*n* = 7)

The hypothalamus contains important sexually dimorphic nuclei, and many of these sex differences are organized by gonadal hormones during the perinatal sensitive period [42, 43]. To identify noncanonical mediators of estrogen-dependent sex-specific neural development, we assayed the miRNA complement of the PN2 hypothalamus by microarray 24 h after females were injected with vehicle (F/Veh), or males were injected with either vehicle (M/Veh) or the aromatase inhibitor, formestane (M/Form). Principal component analysis of the expression of 1407 miRNAs assayed by microarray demonstrated a dramatic effect of sex on the hypothalamic miRNA environment between F/Veh and M/Veh groups at postnatal day 2 (PN2). Orthogonal partial least squares discriminant analysis (OPLS-DA) was used to divide systematic variation in miRNA expression levels into two model components: the predictive (X) component (variation correlated to the factor of interest, e.g., sex) and the orthogonal (Y) component (uncorrelated to the factor of interest). OPLS-DA analysis of the expression of these 1407 miRNAs showed clear separation between male and female miRNA expression profiles along the predictive component (sex) (Fig. 1a), (Q^2^ (cumulative) = 0.429, total amount of variance explained in the x matrix (R^2^X) (cumulative) = 0.449, total amount of variance explained in the y matrix (R^2^Y) (cumulative) = 0.963, P[CV-ANOVA] = 0.0425). A volcano plot-based on this multivariate model demonstrates that a clear majority of miRNAs were downregulated in males relative to females (Fig. 1b). Differential expression analysis of the microarray data, to identify individual sex-biased miRNAs, revealed a significant effect of sex on at least 162 individual miRNAs (FDR ≤ 0.05) (Table 1).

**Table 1:** Expression of neonatal (PN2) hypothalamic miRNAs with statistically significant sex differences

miRNA annotations (mirbase)				Log2 fold change (relative to F/Veh)			
miRNA	Accession	Family	Alignment (mm9)	F/Veh	M/Form	M/Veh	Effect
mmu-let-7a-2	MI0000557	let-7	9:41344799–41344894 (+)	0.0 ± 0.09^A^	−0.103 ± 0.08^AB^	−0.360 ± 0.11^B^	Estrogen (down)
mmu-let-7c-2-3p	MIMAT0005439	let-7	15:85537033–85537127 (+)	0.0 ± 0.06^A^	0.021 ± 0.03^A^	−0.276 ± 0.11^B^	Estrogen (down)
mmu-mir-101a	MI0000148	mir-101	4:101019550–101019632 (−)	0.0 ± 0.07^A^	−0.012 ± 0.11^A^	0.302 ± 0.07^B^	Estrogen (up)
mmu-miR-10b-3p	MIMAT0004538	mir-10	2:74564127–74564194 (+)	0.0 ± 0.07^A^	−0.058 ± 0.09^AB^	−0.303 ± 0.07^B^	Estrogen (down)
mmu-miR-1186	MIMAT0005836		8:32213393–32213514 (−)	0.0 ± 0.14^A^	0.467 ± 0.16^B^	0.866 ± 0.19^B^	Estrogen (up)
mmu-miR-1186b	MIMAT0015644		8:98487444–98487507 (+)	0.0 ± 0.15^A^	0.444 ± 0.14^AB^	0.742 ± 0.21^B^	Other (up)
mmu-miR-1188-3p	MIMAT0017328	mir-1188	12:110850032–110850151 (+)	0.0 ± 0.04^A^	−0.121 ± 0.13^AB^	−0.410 ± 0.13^B^	Estrogen (down)
mmu-miR-1188-5p	MIMAT0005843	mir-1188	12:110850032–110850151 (+)	0.0 ± 0.04^A^	−0.344 ± 0.19^AB^	−0.509 ± 0.11^B^	Estrogen (down)
mmu-miR-1192	MIMAT0005850		19:23223921–23224041 (+)	0.0 ± 0.15^A^	−0.192 ± 0.13^A^	1.134 ± 0.64^B^	Estrogen (up)
mmu-mir-1196	MI0006304		14:62371057–62371175 (−)	0.0 ± 0.06^A^	−0.066 ± 0.11^AB^	−0.359 ± 0.1^B^	Estrogen (down)
mmu-mir-1199	MI0006307		8:86535414–86535532 (−)	0.0 ± 0.21^A^	−0.196 ± 0.1^AB^	−0.551 ± 0.15^B^	Estrogen (down)
mmu-miR-1247-5p	MIMAT0014800	mir-1247	12:111516258–111516339 (−)	0.0 ± 0.18^A^	0.195 ± 0.12^A^	0.618 ± 0.18^B^	Estrogen (up)
mmu-miR-1249-3p	MIMAT0010560	mir-1249	15:84781956–84782053 (−)	0.0 ± 0.14^A^	1.315 ± 0.47^B^	1.405 ± 0.39^B^	Other (up)
mmu-miR-1251-3p	MIMAT0014825	mir-1251	10:91599885–91599968 (−)	0.0 ± 0.1^A^	−0.213 ± 0.13^AB^	−0.447 ± 0.15^B^	Estrogen (down)
mmu-miR-1251-5p	MIMAT0014824	mir-1251	10:91599885–91599968 (−)	0.0 ± 0.18^A^	−0.412 ± 0.16^AB^	−0.486 ± 0.14^B^	Other (down)
mmu-miR-127-3p	MIMAT0000139	mir-127	12:110831056–110831125 (+)	0.0 ± 0.07^A^	−0.116 ± 0.14^AB^	−0.391 ± 0.1^B^	Estrogen (down)
mmu-miR-127-5p	MIMAT0004530	mir-127	12:110831056–110831125 (+)	0.0 ± 0.08^A^	−0.122 ± 0.08^AB^	−0.290 ± 0.03^B^	Estrogen (down)
mmu-mir-134	MI0000160	mir-134	12:110972349–110972419 (+)	0.0 ± 0.06^A^	−0.055 ± 0.07^AB^	−0.290 ± 0.11^B^	Estrogen (down)
mmu-miR-134-5p	MIMAT0000146	mir-134	12:110972349–110972419 (+)	0.0 ± 0.07^A^	−0.207 ± 0.11^AB^	−0.429 ± 0.09^B^	Estrogen (down)
mmu-miR-135a-1-3p	MIMAT0004531	mir-135	9:106056455–106056544 (+)	0.0 ± 0.09^A^	−0.267 ± 0.16^AB^	−0.451 ± 0.13^B^	Other (down)
mmu-miR-135b-5p	MIMAT0000612	mir-135	1:134094665–134094761 (+)	0.0 ± 0.14^A^	−0.340 ± 0.09^A^	−0.809 ± 0.2^B^	Other (down)
mmu-miR-138-1-3p	MIMAT0004668	mir-138	9:122591994–122592092 (+)	0.0 ± 0.04^A^	−0.109 ± 0.14^AB^	−0.217 ± 0.05^B^	Estrogen (down)
mmu-miR-149-5p	MIMAT0000159	mir-149	1:94746955–94747020 (+)	0.0 ± 0.11^A^	0.648 ± 0.24^B^	0.619 ± 0.23^B^	Other (up)
mmu-miR-150-5p	MIMAT0000160	mir-150	7:52377127–52377191 (+)	0.0 ± 0.04^A^	−0.070 ± 0.1^AB^	−0.323 ± 0.11^B^	Estrogen (down)
mmu-miR-15a-5p	MIMAT0000526	mir-15	14:62250864–62250947 (−)	0.0 ± 0.14^A^	−0.154 ± 0.14^AB^	−0.581 ± 0.29^B^	Estrogen (down)
mmu-miR-181b-2-3p	MIMAT0017084	mir-181	2:38709350–38709438 (+)	0.0 ± 0.04^A^	0.240 ± 0.08^AB^	0.320 ± 0.08^B^	Other (up)
mmu-miR-184-3p	MIMAT0000213	mir-184	9:89697098–89697166 (−)	0.0 ± 0.1^A^	−0.613 ± 0.2^B^	−0.759 ± 0.26^B^	Other (down)
mmu-miR-1843b-3p	MIMAT0019346	mir-1843	1:161270489–161270554 (+)	0.0 ± 0.12^A^	0.286 ± 0.08^B^	0.345 ± 0.08^B^	Other (up)
mmu-miR-185-5p	MIMAT0000214	mir-185	16:18327494–18327558 (−)	0.0 ± 0.18^A^	−0.265 ± 0.26^AB^	−0.738 ± 0.12^B^	Estrogen (down)
mmu-mir-188	MI0000230	mir-188	X:6825115–6825182 (−)	0.0 ± 0.07^A^	−0.094 ± 0.09^AB^	−0.349 ± 0.13^B^	Estrogen (down)
mmu-mir-1904	MI0008323		13:110694017–110694096 (+)	0.0 ± 0.07^A^	−0.212 ± 0.09^AB^	−0.268 ± 0.06^B^	Other (down)
mmu-miR-1930-3p	MIMAT0017340		10:77103969–77104052 (+)	0.0 ± 0.1^A^	−0.267 ± 0.11^AB^	−0.384 ± 0.16^B^	Estrogen (down)
mmu-mir-1936	MI0009925		12:103923138–103923230 (−)	0.0 ± 0.05^A^	0.076 ± 0.04^AB^	0.288 ± 0.12^B^	Estrogen (up)
mmu-mir-1938	MI0009927		12:40949299–40949398 (−)	0.0 ± 0.07^A^	−0.174 ± 0.06^AB^	−0.344 ± 0.09^B^	Other (down)
mmu-miR-194-1-3p	MIMAT0016999	mir-194	1:187137198–187137264 (+)	0.0 ± 0.26^A^	−0.073 ± 0.13^A^	−0.517 ± 0.11^B^	Estrogen (down)
mmu-miR-1955-5p	MIMAT0009426		2:92032134–92032231 (+)	0.0 ± 0.08^A^	−0.162 ± 0.08^AB^	−0.413 ± 0.2^B^	Estrogen (down)
mmu-mir-202	MI0000245	mir-202	7:147143588–147143659 (−)	0.0 ± 0.11^A^	−0.391 ± 0.06^B^	−0.456 ± 0.04^B^	Other (down)
mmu-mir-206	MI0000249	mir-1	1:20669091–20669163 (+)	0.0 ± 0.26^A^	−0.331 ± 0.06^AB^	−0.495 ± 0.12^B^	Other (down)
mmu-miR-210-5p	MIMAT0017052	mir-210	7:148407283–148407392 (−)	0.0 ± 0.07^A^	−0.083 ± 0.08^AB^	−0.332 ± 0.12^B^	Estrogen (down)
mmu-mir-214	MI0000698	mir-214	1:164153499–164153608 (+)	0.0 ± 0.16^A^	−0.069 ± 0.1^A^	−0.454 ± 0.15^B^	Estrogen (down)
mmu-miR-216a-5p	MIMAT0000662	mir-216	11:28657012–28657083 (+)	0.0 ± 0.17^A^	−0.189 ± 0.17^AB^	−0.473 ± 0.11^B^	Estrogen (down)
mmu-miR-217-3p	MIMAT0017072	mir-217	11:28663728–28663835 (+)	0.0 ± 0.1^A^	−0.139 ± 0.09^AB^	−0.382 ± 0.1^B^	Estrogen (down)
mmu-mir-218-1	MI0000700	mir-218	5:48615181–48615290 (+)	0.0 ± 0.1^A^	−0.058 ± 0.12^AB^	−0.384 ± 0.1^B^	Other (down)
mmu-miR-218-1-3p	MIMAT0004665	mir-218	5:48615181–48615290 (+)	0.0 ± 0.08^A^	−0.307 ± 0.1^AB^	−0.474 ± 0.16^B^	Other (down)
mmu-miR-219-1-3p	MIMAT0017055	mir-219	17:34161928–34162037 (−)	0.0 ± 0.12^A^	−0.633 ± 0.14^B^	−0.651 ± 0.16^B^	Other (down)
mmu-miR-219-2-3p	MIMAT0017074	mir-219	2:29701151–29701247 (−)	0.0 ± 0.1^A^	−0.063 ± 0.08^A^	−0.394 ± 0.12^B^	Other (down)
mmu-miR-222-5p	MIMAT0017061	mir-221	X:18724019–18724097 (−)	0.0 ± 0.1^A^	−0.293 ± 0.09^B^	−0.393 ± 0.09^B^	Other (down)
mmu-miR-27b-3p	MIMAT0000126	mir-27	13:63402020–63402092 (+)	0.0 ± 0.05^A^	−0.155 ± 0.1^AB^	−0.509 ± 0.17^B^	Other (down)
mmu-miR-28-3p	MIMAT0004661	mir-28	16:24827941–24828026 (+)	0.0 ± 0.04^A^	−0.194 ± 0.05^AB^	−0.298 ± 0.06^B^	Other (down)
mmu-miR-292-3p	MIMAT0000370	mir-290	7:3219190–3219271 (+)	0.0 ± 0.03^A^	−0.217 ± 0.1^AB^	−0.336 ± 0.09^B^	Other (down)
mmu-mir-299	MI0000399	mir-299	12:110948848–110948910 (+)	0.0 ± 0.03^A^	−0.133 ± 0.07^AB^	−0.359 ± 0.06^B^	Other (down)
mmu-miR-299-5p	MIMAT0000377	mir-299	12:110948848–110948910 (+)	0.0 ± 0.12^A^	−0.624 ± 0.21^B^	−0.615 ± 0.22^B^	Other (down)
mmu-miR-3057-3p	MIMAT0014823		10:80734342–80734432 (+)	0.0 ± 0.16^A^	−0.496 ± 0.22^B^	−0.744 ± 0.05^B^	Other (down)
mmu-miR-3059-5p	MIMAT0014811		10:101235326–101235406 (+)	0.0 ± 0.23^A^	−0.340 ± 0.17^AB^	−0.533 ± 0.1^B^	Other (down)
mmu-miR-3060-5p	MIMAT0014826		11:4039367–4039449 (+)	0.0 ± 0.05^A^	−0.159 ± 0.1^AB^	−0.339 ± 0.12^B^	Estrogen (down)
mmu-miR-3061-5p	MIMAT0014828		11:51940248–51940338 (+)	0.0 ± 0.07^A^	−0.456 ± 0.08^B^	−0.705 ± 0.2^B^	Other (down)
mmu-miR-3065-3p	MIMAT0014837	mir-3065	11:119876081–119876167 (+)	0.0 ± 0.09^A^	0.427 ± 0.11^B^	0.602 ± 0.18^B^	Other (up)
mmu-mir-3070a	MI0014032	mir-3070	12:110826153–110826241 (+)	0.0 ± 0.1^A^	−0.270 ± 0.09^AB^	−0.331 ± 0.11^B^	Other (down)
mmu-mir-3070b	MI0014033	mir-3070	12:110826802–110826890 (+)	0.0 ± 0.06^A^	0.075 ± 0.04^A^	−0.290 ± 0.13^B^	Estrogen (down)
mmu-miR-3071-3p	MIMAT0014851		12:110833528–110833607 (−)	0.0 ± 0.07^A^	−0.156 ± 0.06^AB^	−0.350 ± 0.05^B^	Estrogen (down)
mmu-miR-3072-5p	MIMAT0014852		12:110986088–110986170 (+)	0.0 ± 0.18^A^	−0.124 ± 0.13^AB^	−0.489 ± 0.18^B^	Estrogen (down)
mmu-miR-3074-1-3p	MIMAT0014857	mir-3074	13:63402507–63402591 (−)	0.0 ± 0.07^A^	−0.218 ± 0.18^AB^	−0.634 ± 0.29^B^	Estrogen (down)
mmu-miR-3078-3p	MIMAT0014865		14:65210022–65210108 (+)	0.0 ± 0.12^A^	−0.248 ± 0.1^AB^	−0.547 ± 0.26^B^	Other (down)
mmu-miR-3082-3p	MIMAT0014873		17:25968310–25968373 (−)	0.0 ± 0.14^A^	−0.386 ± 0.16^AB^	−0.489 ± 0.12^B^	Other (down)
mmu-miR-3093-3p	MIMAT0014908		3:88019093–88019179 (+)	0.0 ± 0.05^A^	−0.063 ± 0.06^AB^	−0.322 ± 0.15^B^	Estrogen (down)
mmu-miR-3097-5p	MIMAT0014915		5:35363698–35363764 (+)	0.0 ± 0.09^A^	−0.219 ± 0.17^AB^	−0.674 ± 0.24^B^	Estrogen (down)
mmu-miR-30b-5p	MIMAT0000130	mir-30	15:68168977–68169072 (−)	0.0 ± 0.09^A^	−0.115 ± 0.07^AB^	−0.293 ± 0.09^B^	Estrogen (down)
mmu-mir-30c-1	MI0000547	mir-30	4:120442139–120442227 (−)	0.0 ± 0.08^A^	−0.192 ± 0.05^AB^	−0.262 ± 0.06^B^	Other (down)
mmu-miR-31-3p	MIMAT0004634	mir-31	4:88556461–88556566 (−)	0.0 ± 0.06^A^	−0.108 ± 0.11^AB^	−0.382 ± 0.15^B^	Estrogen (down)
mmu-miR-3102-5p	MIMAT0014933		7:108030820–108030923 (−)	0.0 ± 0.15^A^	−0.188 ± 0.19^B^	−0.493 ± 0.22^B^	Estrogen (down)
mmu-miR-3110-3p	MIMAT0014952		X:35563618–35563697 (+)	0.0 ± 0.1^A^	−0.180 ± 0.16^AB^	−0.558 ± 0.24^B^	Estrogen (down)
mmu-miR-32-3p	MIMAT0017050	mir-32	4:56908101–56908170 (−)	0.0 ± 0.16^A^	0.058 ± 0.12^A^	1.341 ± 0.63^B^	Estrogen (up)
mmu-miR-324-3p	MIMAT0000556	mir-324	11:69825545–69825633 (+)	0.0 ± 0.04^A^	−0.004 ± 0.07^A^	−0.300 ± 0.13^B^	Estrogen (down)
mmu-miR-324-5p	MIMAT0000555	mir-324	11:69825545–69825633 (+)	0.0 ± 0.05^A^	−0.140 ± 0.16^AB^	−0.480 ± 0.12^B^	Estrogen (down)
mmu-mir-325	MI0000597	mir-325	X:102574421–102574518 (−)	0.0 ± 0.03^A^	−0.280 ± 0.08^B^	−0.342 ± 0.07^B^	Other (down)
mmu-miR-325-5p	MIMAT0000558	mir-325	X:102574421–102574518 (−)	0.0 ± 0.14^A^	0.350 ± 0.1^AB^	0.606 ± 0.27^B^	Estrogen (up)
mmu-miR-330-3p	MIMAT0000569	mir-330	7:19766814–19766911 (+)	0.0 ± 0.07^A^	−0.159 ± 0.07^AB^	−0.271 ± 0.05^B^	Other (down)
mmu-miR-330-5p	MIMAT0004642	mir-330	7:19766814–19766911 (+)	0.0 ± 0.05^A^	−0.194 ± 0.16^AB^	−0.456 ± 0.13^B^	Estrogen (down)
mmu-miR-337-5p	MIMAT0004644	mir-337	12:110823999–110824095 (+)	0.0 ± 0.05^A^	−0.189 ± 0.12^AB^	−0.453 ± 0.11^B^	Estrogen (down)
mmu-miR-341-5p	MIMAT0017037	mir-341	12:110849710–110849805 (+)	0.0 ± 0.08^A^	−0.001 ± 0.07^A^	−0.370 ± 0.16^B^	Estrogen (down)
mmu-mir-343	MI0005494	mir-343	7:19971992–19972066 (+)	0.0 ± 0.08^A^	−0.143 ± 0.02^AB^	−0.306 ± 0.09^B^	Other (down)
mmu-mir-344-1	MI0000630	mir-344	7:69022656–69022750 (−)	0.0 ± 0.06^A^	−0.049 ± 0.07^A^	−0.310 ± 0.06^B^	Estrogen (down)
mmu-miR-344e-3p	MIMAT0014924	mir-344	7:68880423–68880488 (−)	0.0 ± 0.21^A^	−0.081 ± 0.11^A^	1.116 ± 0.68^B^	Estrogen (up)
mmu-mir-344f	MI0014098	mir-344	7:69191067–69191134 (−)	0.0 ± 0.08^A^	−0.166 ± 0.11^A^	−0.477 ± 0.08^B^	Estrogen (down)
mmu-miR-344f-5p	MIMAT0014931	mir-344	7:69191067–69191134 (−)	0.0 ± 0.11^A^	−0.252 ± 0.15^A^	−0.712 ± 0.13^B^	Estrogen (down)
mmu-miR-3470b	MIMAT0015641	mir-3470	16:44013965–44014090 (+)	0.0 ± 0.18^A^	0.427 ± 0.11^B^	0.410 ± 0.09^B^	Other (up)
mmu-miR-3473d	MIMAT0020632	mir-3473	8:113540350–113540430 (−)	0.0 ± 0.07^A^	−0.177 ± 0.09^AB^	−0.437 ± 0.16^B^	Estrogen (down)
mmu-miR-363-5p	MIMAT0017076	mir-363	X:50094870–50094944 (−)	0.0 ± 0.1^A^	−0.155 ± 0.09^AB^	−0.402 ± 0.07^B^	Estrogen (down)
mmu-mir-370	MI0001165	mir-370	12:110856468–110856546 (+)	0.0 ± 0.08^A^	−0.291 ± 0.11^AB^	−0.394 ± 0.12^B^	Estrogen (down)
mmu-miR-376c-3p	MIMAT0003183	mir-368	12:110960928–110961013 (+)	0.0 ± 0.05^A^	0.101 ± 0.09^AB^	0.280 ± 0.08^B^	Estrogen (up)
mmu-miR-378-3p	MIMAT0003151	mir-378	18:61557489–61557554 (−)	0.0 ± 0.1^A^	−0.550 ± 0.18^B^	−0.818 ± 0.27^B^	Other (down)
mmu-miR-378-5p	MIMAT0000742	mir-378	18:61557489–61557554 (−)	0.0 ± 0.11^A^	−0.246 ± 0.11^AB^	−0.343 ± 0.1^B^	Other (down)
mmu-mir-381	MI0000798	mir-154	12:110965032–110965106 (+)	0.0 ± 0.04^A^	−0.004 ± 0.07^A^	−0.307 ± 0.1^B^	Estrogen (down)
mmu-miR-383-3p	MIMAT0017082	mir-383	8:39315187–39315256 (−)	0.0 ± 0.08^A^	−0.201 ± 0.17^AB^	−0.499 ± 0.19^B^	Estrogen (down)
mmu-miR-383-5p	MIMAT0000748	mir-383	8:39315187–39315256 (−)	0.0 ± 0.07^A^	−0.370 ± 0.18^AB^	−0.706 ± 0.25^B^	Other (down)
mmu-miR-384-3p	MIMAT0001076	mir-384	X:102539621–102539708 (−)	0.0 ± 0.07^A^	0.162 ± 0.07^AB^	0.329 ± 0.09^B^	Estrogen (up)
mmu-mir-412	MI0001164	mir-412	12:110981499–110981578 (+)	0.0 ± 0.07^A^	−0.237 ± 0.09^AB^	−0.421 ± 0.07^B^	Other (down)
mmu-miR-412-5p	MIMAT0017173	mir-412	12:110981499–110981578 (+)	0.0 ± 0.08^A^	−0.353 ± 0.12^B^	−0.497 ± 0.08^B^	Other (down)
mmu-miR-421-5p	MIMAT0017273	mir-95	X:100768260–100768335 (−)	0.0 ± 0.02^A^	−0.310 ± 0.04^B^	−0.322 ± 0.09^B^	Other (down)
mmu-mir-431	MI0001524	mir-431	12:110828657–110828747 (+)	0.0 ± 0.04^A^	−0.187 ± 0.07^AB^	−0.312 ± 0.05^B^	Other (down)
mmu-mir-432	MI0012528	mir-432	12:110833166–110833240 (+)	0.0 ± 0.07^A^	−0.031 ± 0.05^A^	−0.410 ± 0.16^B^	Estrogen (down)
mmu-miR-432	MIMAT0012771	mir-432	12:110833166–110833240 (+)	0.0 ± 0.09^A^	−0.097 ± 0.13^AB^	−0.438 ± 0.18^B^	Estrogen (down)
mmu-mir-433	MI0001525	mir-433	12:110829925–110830048 (+)	0.0 ± 0.07^A^	−0.127 ± 0.05^AB^	−0.258 ± 0.05^B^	Other (down)
mmu-miR-452-5p	MIMAT0001637	mir-452	X:69507563–69507647 (−)	0.0 ± 0.09^A^	0.060 ± 0.09^A^	−0.316 ± 0.09^B^	Estrogen (down)
mmu-mir-465c-1	MI0005500	mir-465	X:64079130–64079210 (−)	0.0 ± 0.14^A^	0.500 ± 0.22^AB^	0.986 ± 0.39^B^	Estrogen (up)
mmu-mir-465c-2	MI0005501	mir-465	X:64085692–64085772 (−)	0.0 ± 0.11^A^	0.434 ± 0.2^AB^	1.022 ± 0.4^B^	Estrogen (up)
mmu-miR-466 h-5p	MIMAT0004884	mir-467	2:10436518–10436598 (+)	0.0 ± 0.19^A^	0.235 ± 0.09^AB^	0.451 ± 0.18^B^	Estrogen (up)
mmu-miR-466 l-5p	MIMAT0017322	mir-467	2:10437724–10437844 (+)	0.0 ± 0.06^A^	−0.250 ± 0.06^AB^	−0.314 ± 0.11^B^	Other (down)
mmu-mir-467a-9	MI0014074	mir-467	2:10420020–10420102 (+)	0.0 ± 0.03^A^	−0.383 ± 0.11^B^	−0.522 ± 0.16^B^	Other (down)
mmu-mir-467b	MI0004671	mir-467	2:10402875–10402947 (+)	0.0 ± 0.07^A^	−0.342 ± 0.14^AB^	−0.459 ± 0.15^B^	Other (down)
mmu-mir-467c	MI0005512	mir-467	2:10395558–10395654 (+)	0.0 ± 0.14^A^	−0.289 ± 0.06^AB^	−0.358 ± 0.11^B^	Other (down)
mmu-miR-468-3p	MIMAT0002109		6:81846593–81846670 (−)	0.0 ± 0.08^A^	−0.157 ± 0.09^AB^	−0.548 ± 0.27^B^	Estrogen (down)
mmu-mir-490	MI0005002	mir-490	6:36371742–36371825 (+)	0.0 ± 0.09^A^	−0.332 ± 0.07^B^	−0.402 ± 0.09^B^	Other (down)
mmu-miR-490-5p	MIMAT0017261	mir-490	6:36371742–36371825 (+)	0.0 ± 0.1^A^	−0.244 ± 0.13^AB^	−0.414 ± 0.12^B^	Other (down)
mmu-miR-497-5p	MIMAT0003453	mir-497	11:70048219–70048302 (+)	0.0 ± 0.14^A^	0.346 ± 0.14^AB^	0.414 ± 0.11^B^	Other (up)
mmu-mir-500	MI0004702	mir-500	X:6814809–6814900 (−)	0.0 ± 0.06^A^	−0.031 ± 0.1^A^	−0.369 ± 0.17^B^	Estrogen (down)
mmu-miR-504-3p	MIMAT0017277	mir-504	X:56350835–56350913 (−)	0.0 ± 0.05^A^	−0.370 ± 0.16^AB^	−0.493 ± 0.21^B^	Other (down)
mmu-miR-504-5p	MIMAT0004889	mir-504	X:56350835–56350913 (−)	0.0 ± 0.15^A^	0.126 ± 0.05^A^	0.421 ± 0.1^B^	Estrogen (up)
mmu-mir-5098	MI0018006	mir-1954	5:77701782–77701863 (+)	0.0 ± 0.17^A^	0.208 ± 0.09^AB^	0.488 ± 0.1^B^	Estrogen (up)
mmu-mir-5100	MI0018008		11:60542165–60542228 (+)	0.0 ± 0.07^A^	0.323 ± 0.04^B^	0.254 ± 0.07^B^	Other (up)
mmu-miR-5109	MIMAT0020617		5:17282567–17282653 (−)	0.0 ± 0.05^A^	0.381 ± 0.09^B^	0.298 ± 0.09^B^	Other (up)
mmu-miR-5114	MIMAT0020622		19:44377661–44377721 (+)	0.0 ± 0.1^A^	−0.183 ± 0.1^AB^	−0.476 ± 0.12^B^	Estrogen (down)
mmu-miR-5115	MIMAT0020623		2:72850911–72850984 (−)	0.0 ± 0.15^A^	0.297 ± 0.1^AB^	0.580 ± 0.2^B^	Estrogen (up)
mmu-miR-5124	MIMAT0020634		13:40961160–40961231 (+)	0.0 ± 0.07^A^	−0.249 ± 0.08^AB^	−0.277 ± 0.06^B^	Other (down)
mmu-miR-5131	MIMAT0020642		14:46277636–46277728 (−)	0.0 ± 0.04^A^	−0.067 ± 0.07^A^	−0.353 ± 0.15^B^	Other (down)
mmu-miR-540-3p	MIMAT0003167	mir-540	12:110824290–110824356 (+)	0.0 ± 0.03^A^	−0.162 ± 0.08^AB^	−0.415 ± 0.11^B^	Estrogen (down)
mmu-mir-543	MI0003519	mir-329	12:110955468–110955543 (+)	0.0 ± 0.09^A^	−0.189 ± 0.06^AB^	−0.329 ± 0.09^B^	Other (down)
mmu-miR-592-5p	MIMAT0003730	mir-592	6:27886655–27886750 (−)	0.0 ± 0.08^A^	−0.247 ± 0.11^AB^	−0.496 ± 0.11^B^	Other (down)
mmu-mir-615	MI0005004	mir-615	15:102845341–102845432 (+)	0.0 ± 0.15^A^	−0.315 ± 0.09^AB^	−0.542 ± 0.11^B^	Other (down)
mmu-miR-652-3p	MIMAT0003711	mir-652	X:139173543–139173640 (+)	0.0 ± 0.07^A^	−0.394 ± 0.24^AB^	−0.652 ± 0.18^B^	Other (down)
mmu-miR-652-5p	MIMAT0017260	mir-652	X:139173543–139173640 (+)	0.0 ± 0.05^A^	−0.025 ± 0.08^A^	−0.371 ± 0.15^B^	Estrogen (down)
mmu-miR-665-5p	MIMAT0017238	mir-665	12:110824524–110824617 (+)	0.0 ± 0.12^A^	−0.205 ± 0.19^AB^	−0.531 ± 0.15^B^	Estrogen (down)
mmu-miR-666-3p	MIMAT0004823	mir-666	12:110955295–110955393 (+)	0.0 ± 0.11^A^	0.040 ± 0.11^A^	−0.401 ± 0.17^B^	Estrogen (down)
mmu-miR-666-5p	MIMAT0003737	mir-666	12:110955295–110955393 (+)	0.0 ± 0.05^A^	−0.097 ± 0.12^AB^	−0.351 ± 0.13^B^	Estrogen (down)
mmu-mir-668	MI0004134	mir-668	12:110972942–110973007 (+)	0.0 ± 0.06^A^	−0.208 ± 0.1^AB^	−0.369 ± 0.1^B^	Other (down)
mmu-miR-668-5p	MIMAT0017237	mir-668	12:110972942–110973007 (+)	0.0 ± 0.07^A^	−0.235 ± 0.14^AB^	−0.502 ± 0.11^B^	Estrogen (down)
mmu-mir-669a-4	MI0014054	mir-467	2:10400942–10401028 (+)	0.0 ± 0.09^A^	−0.160 ± 0.08^AB^	−0.325 ± 0.06^B^	Other (down)
mmu-miR-669b-5p	MIMAT0003476	mir-467	2:10389417–10389513 (+)	0.0 ± 0.09^A^	−0.334 ± 0.06^A^	0.503 ± 0.18^B^	Estrogen (up)
mmu-miR-669 h-3p	MIMAT0005842	mir-467	2:10439782–10439906 (+)	0.0 ± 0.21^A^	−0.233 ± 0.17^AB^	−0.655 ± 0.26^B^	Estrogen (down)
mmu-miR-669 l-3p	MIMAT0017345	mir-467	2:10390598–10390695 (+)	0.0 ± 0.09^A^	0.203 ± 0.23^AB^	0.639 ± 0.22^B^	Estrogen (up)
mmu-miR-670-3p	MIMAT0017242	mir-670	2:94101457–94101556 (−)	0.0 ± 0.03^A^	−0.336 ± 0.11^B^	−0.333 ± 0.06^B^	Other (down)
mmu-miR-671-3p	MIMAT0004821	mir-671	5:24097932–24098029 (+)	0.0 ± 0.1^A^	−0.241 ± 0.15^A^	−0.779 ± 0.25^B^	Estrogen (down)
mmu-mir-673	MI0004601	mir-673	12:110810200–110810290 (+)	0.0 ± 0.07^A^	−0.220 ± 0.07^AB^	−0.347 ± 0.13^B^	Other (down)
mmu-miR-673-3p	MIMAT0004824	mir-673	12:110810200–110810290 (+)	0.0 ± 0.07^A^	−0.394 ± 0.14^AB^	−0.500 ± 0.24^B^	Other (down)
mmu-miR-679-3p	MIMAT0017248		12:110953787–110953860 (+)	0.0 ± 0.09^A^	−0.429 ± 0.08^B^	−0.434 ± 0.18^B^	Other (down)
mmu-miR-679-5p	MIMAT0003455		12:110953787–110953860 (+)	0.0 ± 0.07^A^	−0.239 ± 0.13^AB^	−0.367 ± 0.11^B^	Other (down)
mmu-mir-683-2	MI0010690	mir-683	13:50696341–50696449 (−)	0.0 ± 0.06^A^	−0.236 ± 0.1^AB^	−0.444 ± 0.12^B^	Other (down)
mmu-miR-691	MIMAT0003470		16:74342235–74342312 (−)	0.0 ± 0.17^A^	0.063 ± 0.14^A^	0.731 ± 0.18^B^	Estrogen (up)
mmu-miR-700-5p	MIMAT0017256		4:134972470–134972548 (−)	0.0 ± 0.07^A^	−0.252 ± 0.09^AB^	−0.435 ± 0.13^B^	Other (down)
mmu-mir-702	MI0004686	mir-702	5:137467303–137467411 (+)	0.0 ± 0.09^A^	−0.414 ± 0.12^B^	−0.359 ± 0.1^B^	Estrogen (down)
mmu-mir-707	MI0004691		7:52105069–52105141 (+)	0.0 ± 0.06^A^	−0.337 ± 0.05^B^	−0.362 ± 0.07^B^	Other (down)
mmu-mir-758	MI0004129	mir-379	12:110951020–110951100 (+)	0.0 ± 0.04^A^	−0.251 ± 0.07^AB^	−0.353 ± 0.13^B^	Estrogen (down)
mmu-miR-762	MIMAT0003892	mir-762	4:108690260–108690335 (+)	0.0 ± 0.23^A^	0.089 ± 0.12^AB^	0.485 ± 0.17^B^	Estrogen (up)
mmu-miR-873-5p	MIMAT0004936	mir-873	4:36615543–36615619 (−)	0.0 ± 0.12^A^	−0.367 ± 0.07^B^	−0.448 ± 0.11^B^	Other (down)
mmu-mir-874	MI0005479	mir-874	13:58124486–58124561 (−)	0.0 ± 0.1^A^	−0.034 ± 0.07^A^	−0.300 ± 0.09^B^	Estrogen (down)
mmu-miR-874-3p	MIMAT0004853	mir-874	13:58124486–58124561 (−)	0.0 ± 0.08^A^	−0.247 ± 0.11^A^	−0.613 ± 0.17^B^	Estrogen (down)
mmu-miR-874-5p	MIMAT0017268	mir-874	13:58124486–58124561 (−)	0.0 ± 0.11^A^	−0.104 ± 0.09^A^	−0.515 ± 0.15^B^	Estrogen (down)
mmu-miR-877-5p	MIMAT0004861	mir-877	17:36097675–36097759 (−)	0.0 ± 0.07^A^	0.129 ± 0.13^A^	−0.360 ± 0.09^B^	Estrogen (down)
mmu-mir-92a-1	MI0000719	mir-25	14:115443649–115443728 (+)	0.0 ± 0.08^A^	−0.063 ± 0.07^AB^	−0.283 ± 0.08^B^	Estrogen (down)
mmu-miR-93-5p	MIMAT0000540	mir-17	5:138606751–138606838 (−)	0.0 ± 0.03^A^	−0.101 ± 0.09^AB^	−0.311 ± 0.09^B^	Estrogen (down)
mmu-mir-96	MI0000583	mir-96	6:30119446–30119551 (−)	0.0 ± 0.05^A^	0.116 ± 0.08^A^	−0.282 ± 0.1^B^	Estrogen (down)
mmu-miR-98-3p	MIMAT0017023	let-7	X:148347757–148347864 (+)	0.0 ± 0.19^A^	0.290 ± 0.13^AB^	0.511 ± 0.18^B^	Estrogen (up)

Different levels of superscript letter indicates statistically significant effect (false discovery rate ≤ 0.05)

Sex differences in gene expression during this period are the combined product of chromosomal and gonadal hormone effects. To determine the role of organizational estrogen in this sex-specific miRNA regulation, we examined the impact of aromatase inhibition on the neonatal hypothalamus miRNA environment. A multivariate model of the expression of the 162 sex-biased miRNAs identified above confirmed this dysmasculinization. OPLS-DA showed significant separation between the three groups along the predictive component (treatment group) (Q^2^ (cumulative) = 0.39, total amount of variance explained in the x matrix (R^2^X) (cumulative) = 0.567, total amount of variance explained in the y matrix (R^2^Y) (cumulative) = 1, P[CV-ANOVA] = 0.060). A plot of this model (Fig. 1c) shows that the F/Veh group is clustered around one central component, while the M/Form group is a distinct intermediary between male and female vehicle groups.

Consistent with our previous work, a subset of sex-biased miRNAs was characterized as estrogen-responsive based on the extent this sex-biased pattern of expression was disrupted by formestane. The extent of the dysmasculinization resulting from disrupting estrogen signaling can be seen in Fig. 1d, where the mean M/Form expression of individual sex-biased miRNAs is plotted on a continuum between the average expression of these M/Veh and F/Veh groups [M/Form expression on continuum = (M/Form−F/Veh)/(M/Veh−F/Veh)]. While the magnitude of the basal sex difference varied between the 162 miRNAs, M/Form expression levels of 92 of these miRNAs was closer to F/Veh than M/Veh levels. The susceptibility of these 92 miRNAs to the dysmasculinizing effects of aromatase inhibition suggests that they are responsive to estrogen. Seventy-one of these estrogen-responsive miRNAs were reduced in M/Veh relative to F/Veh, likely suppressed by estrogen, while 21 miRNAs were elevated (Fig. 1e). The sex-biased expression of the remaining 70 miRNAs were resistant to formestane disruption, suggesting their expression may be dependent on sex chromosome complement, though the influence of androgens cannot be excluded.

### Estrogen regulation of clustered miRNA genes on chromosome 12

**Fig. 2:**
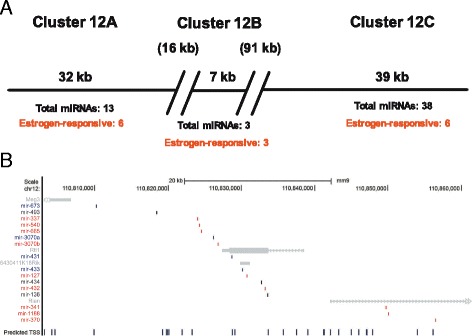
Estrogen regulation of clustered miRNA genes on chromosome 12. **a** Twenty-four sex-biased miRNAs are encoded in three clusters (designated 12A, 12B, and 12C) within a ~ 175 kb region of chromosome 12 [mm9 chr12 110810200–110986170 (+)]. These 24 miRNAs constitute 44% of the miRNAs located in these clusters, which is well above the background rate of 16% of the total clustered miRNAs we assayed that were sex-biased. **b** This effect is even more impressive when focusing on 12A and 12B. Nine of 16 miRNAs in 12A and 12B were estrogen-responsive (*colored red*), and an additional four were characterized as “other” (*colored blue*). Together, 81% of the miRNAs in these two clusters are reduced in control males relative to females, suggesting the miRNA genes in this locus are co-regulated. Ten transcription start sites (TSSs) have been mapped just to the 12A locus; thus, it is unlikely that they are regulated from a shared promoter. Instead, it appears that estrogen could be affecting the expression of these miRNAs through the epigenetic regulation of the entire locus. miRNA clusters were defined by MetaMirCluster using a maximum inter-miRNA distance of 10 kb [34]. TSS mapping data was produced by the FANTOM5 Consortium and obtained as a UCSC Genome Browser public track (FANTOM5 TSS peaks [robust]) [35]

Within the mouse genome, approximately 30% of miRNA genes are located in clusters [34]. These clustered miRNAs are often co-expressed. Some co-expressed miRNAs are processed from a shared polycistronic primary transcript; while other clusters of miRNAs respond to epigenetic modifications in a shared local chromatin structure [44, 45]. To screen for genetic loci that may be epigenetically programmed at this level, we examined the genomic distribution of sex-biased miRNAs to identify those that were in close proximity to each other. miRNA clusters were defined using MetaMirCluster with a maximum inter-miRNA distance of 10 kb [34]. We determined that 24 sex-biased miRNAs were encoded in three clusters located within an approximately 175 kb region of chromosome 12 (Fig. 2a). For clarity, we have designated these clusters: 12A [miR-673_miR-136, mm9 chr12 110810200–110833598 (+)], 12B [miR-341_miR-370, mm9 chr12 110849710–110856546 (+)] and 12C [miR-379_miR-3072, mm9 chr12 110947270–110986170(+)]. These 24 miRNAs constitute 44% of the miRNAs in these clusters, well above the background rate of 16% of total sex-biased miRNAs that were located in clusters. This effect is even more impressive when focusing on 12A and 12B (Fig. 2b). Thirteen of 16 miRNAs in 12A and 12B were sex-biased, and 9 of these appeared to be estrogen-responsive. Together, 81% of the miRNAs in these two clusters were reduced in control males relative to females, suggesting the miRNA genes in this locus are co-regulated. Based on transcription start site mapping by the FANTOM5 Consortium, there are 10 predicted TSSs just within the 12A cluster; thus, it is unlikely that these miRNAs are regulated from a shared promoter [35]. Instead, it appears that estrogen could be affecting the expression of these miRNAs through epigenetic regulation of the entire locus.

### Transcriptome-wide mapping of Argonaute footprints by Ago HITS-CLIP

**Fig. 3:**
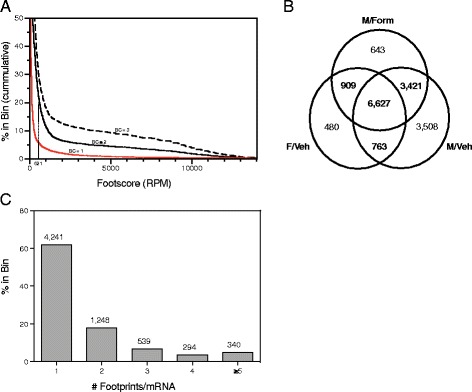
Ago HITS-CLIP empirically identifies robust Argonaute (Ago) footprints across the neonatal (PN2) hypothalamus transcriptome. **a** A histogram of the mean footscore [reads per million (RPM)] of each distinct Ago footprint that aligned to RefSeq mRNAs revealed a wide range and long-tailed distribution of footscores, with a high proportion of footprints having low footscores (Q1, 24; median, 78; Q3, 233; mean, 486). Footprints with high footscores were more likely to be identified in more than one group (biological complexity [BC] ≥ 2). We focused on a subset of footprints with footscores above the 80th percentile (footscore > 621). 16,351 Ago footprints met this criterion. **b** A Venn diagram of the distribution of these footprints across biological groups shows that 72% were present in more than one group (BC ≥ 2). There is also a qualitative effect of estrogen on the Ago footprint population, with the M/Form group sharing more footprints with F/Veh (59% shared) and M/Veh (63%) than F/Veh and M/Veh groups share with each other (47%). Ago footprints with a mean footscore > 621 and a BC ≥ 2 were considered “robust” and used in downstream analyses (indicated by *gray shading*). **c** A histogram of the number of robust Ago footprints aligning to each of 6689 distinct mRNAs shows that 48% of mRNAs contain more than one footprint, with an average of 1.8 footprints/mRNA. Sixteen percent of targeted mRNAs contain three or more footprints

To empirically identify genes under miRNA-mediated regulation, we performed Ago HITS-CLIP analysis of the PN2 hypothalamus. A total of 147,424 unique Ago footprints aligning to RefSeq annotated mRNA were identified across our three treatment groups: 54,573 of these were present in F/Veh, 77,338 in M/Form, and 101,046 in M/Veh. Two criteria were used to identify robust Ago footprints for downstream analyses. The first, footscore, was the average number of reads [reads per million (RPM)] for each footprint across treatment groups. The second, biological complexity (BC), was the number of treatment groups in which the footprint was present. This measure, adapted from the original Ago HITS-CLIP study, was used to establish reproducibility for what was essentially a binary outcome; an Ago footprint was either present or absent in a treatment group [24]. A histogram of the average footscore for each Ago footprint revealed a wide range (foot score min: 11; max: 453,200) and an extremely long-tailed distribution, with a high proportion of footprints having low footscores (Q1 24; median 78; Q3 233; mean 486) (Fig. 3a). Importantly, Ago footprints with high footscores were more likely to be identified in more than one group (BC ≥ 2), indicating that footprints with higher footscores are more robust. Examining this distribution, we focused on 16,351 footprints with footscores above 621 (the 80th percentile of footprints with BC ≥ 2 demarcated in Fig. 3a). Seventy-two percent of these footprints (11,720) were shared by more than one group (BC ≥ 2) and were considered “robust” and used in downstream analyses (Fig. 3b). Consistent with the intermediate miRNA expression phenotype resulting from aromatase inhibition, the M/Form group shared 59 and 63% of robust Ago footprints with F/Veh and M/Veh, respectively, while F/Veh and M/Veh groups shared only 47%. The 11,720 robust footprints were present in mRNAs encoded by 6689 distinct genes, and 48% of these mRNAs contained more than one footprint, with an average of 1.8 Ago footprints/transcript (Fig. 3c).

### Sex-biased miRNA regulate networks of genes relevant to sexual differentiation of the brain

**Fig. 4:**
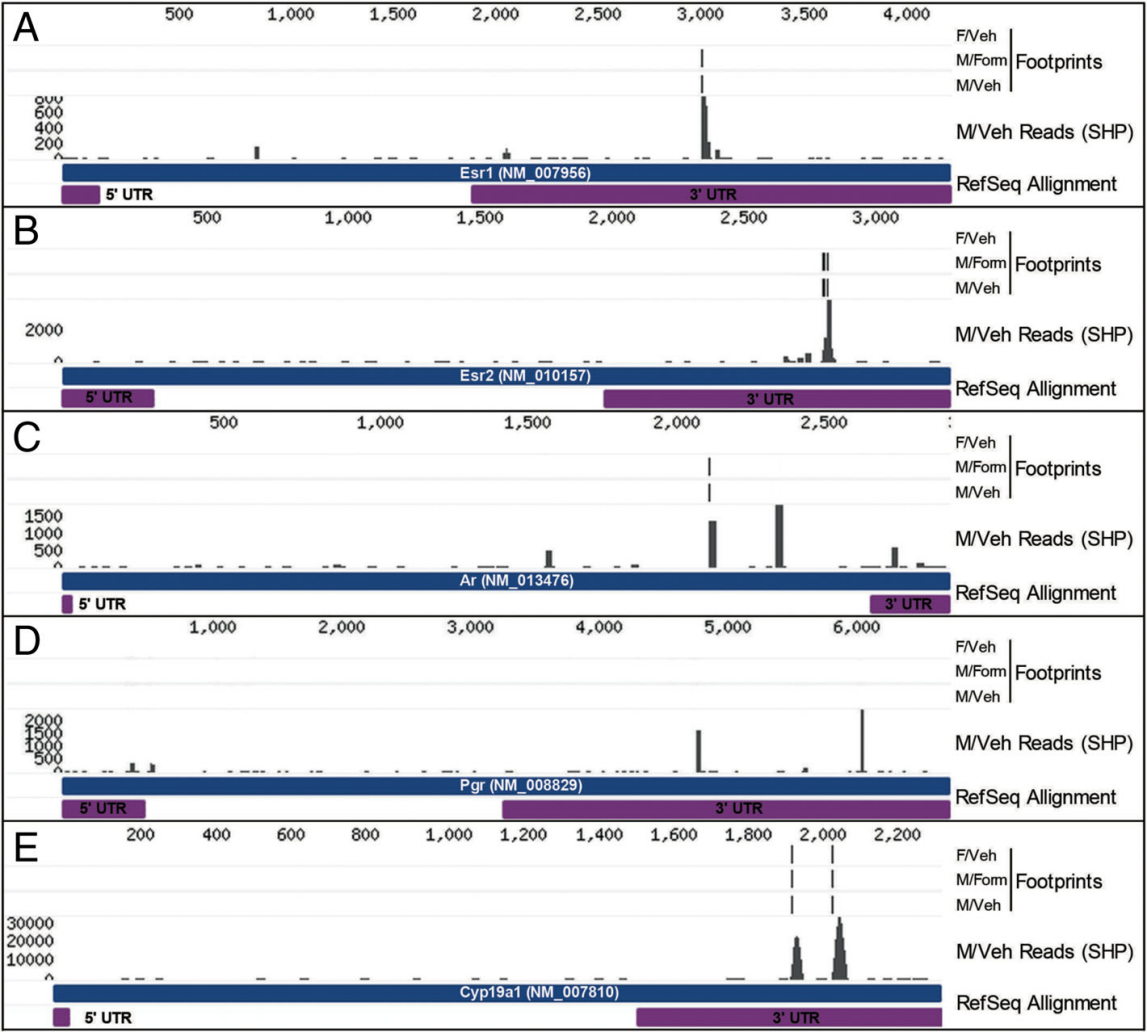
Robust argonaute (Ago) footprints are present in the mRNAs of genes that mediate steroid signaling in the neonatal (PN2) hypothalamus. The distribution of Ago footprints in Esr1 (**a**), Esr2 (**b**), Ar (**c**), Pgr (**d**), and Cyp19a1 (**e**) are shown. The vertical bars in the first three tracks indicate robust Ago footprints (footscore > 621, BC ≥ 2) in F/Veh, M/Form, or M/Veh groups. 5′ and 3′ UTRs annotated in GenBank RefSeq RNA features are indicated in purple. The majority of Ago footprints aligned to the target mRNA’s 3′ UTR. A stacked height profile (SHP) indicates the read counts of overlapping oligos that aligned to the designated mRNA in the M/Veh group

To determine if miRNAs play a role in mediating the effects of organizational hormones during the perinatal sensitive period, we examined the distribution of Ago footprints across mRNAs involved in steroid signaling in the PN2 hypothalamus. Robust Ago footprints (average footscore ≥ 621 and biological complexity ≥ 2) were present in transcripts encoding estrogen receptor α (*Esr1*), estrogen receptor β (*Esr2*), androgen receptor (*Ar*), and aromatase (*Cyp19a1*), though not in progesterone receptor (*Pgr*) (Fig. 4). In addition, the footprints in *Esr1*, *Esr2*, and *Cyp19a1* were located in the 3′ UTR. Finally, according to our HITS-CLIP analysis, most of these footprints are targeted by one or more sex-biased miRNA (Additional file 1: Table S1). Surprisingly, no footprints were identified in the estrogen-responsive hypothalamic hormones oxytocin, arginine vasopressin, or corticotropin-releasing factor.

**Fig. 5:**
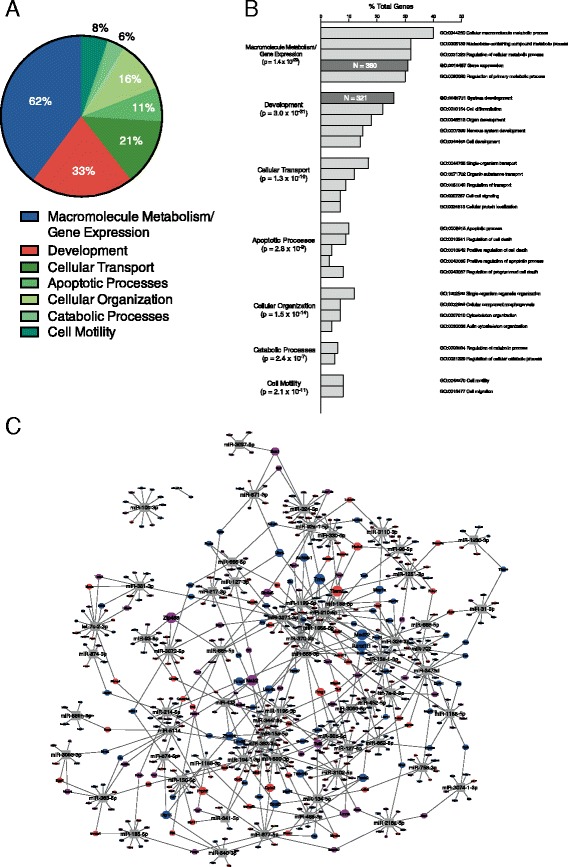
Estrogen-downregulated miRNAs target a network of genes enriched for biological processes relevant to sexual differentiation of the brain. **a** Limiting the number of targets per miRNA to no more than the top 25 (based on footscore), we interrogated a list of 1252 top genes targeted by estrogen-downregulated miRNAs for over-represented terms. ClueGO identified 7 clusters of 132 significantly enriched GO terms (Biological Processes). Cluster size in the pie graph is determined by the proportion of total GO term-gene annotations collapsed within a cluster. **b** The percentage of gene targets annotated by a clustered GO term. **c** Two GO terms, Gene Expression and System Development, seem particularly relevant to sexual differentiation of the brain. Five hundred and forty-five targets of estrogen-downregulated miRNAs were annotated with one or both of these GO terms and were used to generate a network. This network consists of estrogen-downregulated miRNAs and their target genes as nodes, and the miRNA-target connections as edges to allow the visualization of these interactions at a broad level. Genes annotated only with Gene Expression (*n* = 224) are colored *blue*, genes annotated only with System Development (*n* = 224) are colored *red*, and genes annotated with both terms (*n* = 156) are colored *purple*. Twenty-five percent of these genes are targeted by more than one miRNA. The size of individual gene nodes is proportional to the number of miRNAs targeting that gene, and ranges from one to four miRNAs/gene

A common strategy used to determine the biological role of groups of miRNAs involves asking if the genes they target converge on specific biological processes. To test the hypothesis that estrogen-responsive miRNAs regulate gene networks relevant to sexual differentiation of the brain, we looked for gene ontology (GO) terms enriched in Ago HITS-CLIP identified targets of estrogen-downregulated miRNAs, as the expression of their gene targets should therefore be increased. GO terms are organized hierarchically, and individual terms can be constituents of multiple parent categories. Therefore, we used ClueGO to cluster-enriched terms into functionally related groups. We limited the number of targets per miRNA to no more than 25, as ranked by the footscore of the footprint targeted by the individual miRNA. We established this limit as we found that there was a wide range in the number of identified genes targeted by individual miRNA (12 to >100), and we wanted to avoid the broad targets of a limited number of promiscuous miRNAs to overwhelm the impact of the complete set of estrogen-downregulated miRNAs. Interrogating a list of 1252 unique genes targeted by estrogen-downregulated miRNA, ClueGO identified 7 clusters of 132 significantly enriched GO terms (Biological Processes) (Fig. 5a, b). Cluster names were adapted from the GO term within each cluster annotating the largest number of genes. These GO clusters (with cluster *p* value corrected for multiple comparisons by Bonferroni step-down) were macromolecule metabolism/gene expression (*p* = 1.4 × 10^–29)^, development (*p* = 3.0 × 10^−31^), cellular transport (*p* = 1.3 × 10^−10^), apoptotic processes (*p* = 2.8 × 10^−9^), cellular organization (*p* = 1.5 × 10^−14^), catabolic processes (*p* = 2.4 × 10^−7^), and cell motility (*p* = 2.1 × 10^−11^) [39]. Because individual genes are annotated with multiple GO terms, and after collapsing individual GO term-gene annotations within GO clusters, a total of 2111 GO cluster-gene annotations of 1216 distinct genes were made. The remaining 36 miRNA targets were not annotated with any of the clustered GO terms. Seven hundred and eighty genes were annotated with the macromolecule metabolism/gene expression cluster (62% of total), 408 genes with the development cluster (33%), 264 with the cellular transport cluster (21%), 134 with the apoptotic processes cluster (11%), 203 with the cellular organization cluster (16%), 69 with the catabolic processes cluster (6%), and 103 with the cell motility cluster (8%).

Two of the GO terms that were highly enriched within the set of Ago HITS-CLIP identified targets of estrogen-downregulated miRNAs seemed particularly relevant to sexual differentiation of the brain: Gene Expression (GO:0010467) and System Development (GO:0048731). We generated an interaction network to visualize these miRNA-target relationships at a broad level, using the networking program Cytoscape (Fig. 5c). Estrogen-downregulated miRNAs and the subset of their target genes annotated with these GO terms served as the nodes of this network, while Ago HITS-CLIP identified connections served as the edges. The initial layout of this network was generated using the program’s default “organic” algorithm, in which nodes are modeled as mutually repulsive objects, edges induce attractive forces between the nodes they join, then nodes are placed to minimized the sum of these forces. This algorithm tends to result in the clustering of tightly connected nodes, which can be functionally related, though the final layout of our network was altered slightly to limit visual interference amongst nodes. In total, 545 targets of estrogen-downregulated miRNAs (45% of the complete set) were annotated with one or both of these GO terms; 224 with only Gene Expression, 165 with only System Development, and 156 with both terms. Individual gene nodes were sized in proportion to the number of miRNAs targeting that gene and ranged from 1 to 4 miRNAs/genes. Most target gene nodes were connected to only one estrogen-downregulated miRNA, though 135 (25%) were targets of two or more.

## Discussion

### Sex differences in the miRNA environment of the neonatal hypothalamus

Biological sex is a strong predictor of many aspects of neurodevelopmental disorders, including incidence, presentation, and therapeutic outcomes [2]. This is likely the product of interactions between risk factors, such as genetic background or environmental insults, and sex-specific development [42, 43, 46]. Many known sex differences in the brain are organized by exposure to a rise in testosterone in males during the perinatal-sensitive period. In appropriate cell populations, this testosterone is converted to estrogen by a neuronal aromatase, altering gene expression patterns to masculinize and defeminize neurocircuitry and directing the development of a neural substrate that can respond appropriately to the adult hormonal environment [5, 6]. While the primary effector, estrogen, is shared, the cellular processes responsible for this divergent development vary widely across brain regions [11]. miRNAs, with their ability to dynamically regulate the expression of hundreds of genes, may be an exciting and novel regulatory mechanism poised to translate this estrogen signal into brain region-specific responses.

To test this hypothesis, we examined the miRNA content of the neonatal hypothalamus. The hypothalamus contains sexually dimorphic structures responsible for driving sex differences in behavior and physiology, many of which are organized by estrogen during the perinatal-sensitive period [47]. Principal component analysis of the expression of 1407 miRNAs assayed by microarray demonstrated dramatic and robust sex difference in the hypothalamic miRNA environment by postnatal day 2. The increased variability observed in M/Veh samples supports the dynamic nature of this developmental window, when differences in sampling of only hours can have a profound impact on observations. A volcano plot based on the above model showed that a clear majority of miRNAs was downregulated in the M/Veh group. Based on the negative effect miRNAs have on their target’s expression, it is interesting to speculate that this indicates an overall relief of the baseline inhibition of cellular processes responsible for the sexual differentiation of the brain. Though the observation of the effect of sex at such a broad level is quite impressive, it is certainly not the case that all of these miRNAs are involved in sexual differentiation. Therefore, we performed differential expression analysis and identified 162 individual miRNAs with statistically significant sex differences in their expression (Table 1). Several of these miRNAs (mir-299, mir-431, mir-467c, mir-222, mir-32, mir-330, mir-384, mir-665, and mir-671) have previously been identified as sex-biased in the neonatal mouse whole brain and/or rat cortex [23, 48].

Sex differences in gene expression during this period are the combined product of chromosomal and gonadal hormone effects. To determine the role of organizational estrogen in this sex-specific miRNA regulation, we examined the impact of aromatase inhibition on the expression of sex-biased miRNA in the neonatal hypothalamus. In agreement with our previous findings in the whole brain, disruption of this expression pattern was evident in males 24 h following a single PN1 injection of the aromatase inhibitor, formestane [23]. A multivariate model of the expression of the 162 differentially expressed miRNAs showed clear separation of all three groups, with samples from formestane-treated males (M/Form) as a distinct intermediary between male and female vehicle-treated groups. This intermediate expression profile is evidence that not all of the observed sex differences are driven by estrogen. In a process consistent with our previous study, a subset of sex-biased miRNAs was characterized as estrogen-responsive based on the extent this sex-biased pattern of expression was disrupted by formestane [23]. While the magnitude of the basal sex difference varied between the 162 miRNAs, the M/Form expression of 92 of these miRNAs was closer to F/Veh than M/Veh levels; therefore, these 92 miRNAs were categorized as estrogen-responsive.

### Estrogen regulation of miRNA genes clustered on chromosome 12

It has been previously suggested that the persistence of estrogen’s organizational effects into adulthood, despite the transient nature of the perinatal testosterone surge, supports a role for epigenetic programming in sexual differentiation of the brain [49]. Indeed, estrogen-dependent sex differences in the DNA methylation or the distribution of histone modifications have been identified both at the level of individual gene promoters and more broadly genome-wide [50–52]. Unfortunately, due in large part to rapid processing of primary miRNA transcripts into stem-loop precursor miRNA and the resulting difficulty in defining the transcriptional start sites and promoters of miRNAs genes, the characterization of their transcriptional regulation has lagged behind our understanding of the function of their mature miRNA products. Still, the majority of miRNA genes are intergenic and transcribed independent of protein-coding genes. This transcription is RNA polymerase II-dependent and thought to be regulated by the same mechanisms utilized by other genes [53, 54].

Within the mouse genome, approximately 30% of miRNA genes are located in clusters [34]. The expression of clustered miRNAs up to 50 kb apart are highly correlated [45]. This co-expression can result from their being processed from single polycistronic primary transcripts or from changes in a shared local chromatin environment through other epigenetic modifications [44, 45]. Lessons from estrogen signaling in other contexts have highlighted the need to think beyond the classic interactions of steroid hormones, their receptors, and response elements in proximal gene promoters [22, 55, 56]. This seems to be particularly true for genes that are transcriptionally suppressed by estrogen. Whereas estrogen-upregulated genes are enriched for *Esr1* binding at proximal estrogen response elements, downregulated genes are not [22, 56]. Instead, interactions between multiple estrogen receptors, or estrogen receptors and cofactors, can occur distally, generating chromatin looping structures and partitioning affected genes into genomic subregions under shared transcriptional suppression [55].

To identify loci that may be epigenetically programmed at this level, we looked for sex-biased miRNAs encoded in close proximity to each other. Interestingly, 24 sex-biased miRNAs are located in three clusters encoded within an approximately 175-kb region of chromosome 12, which we have designated 12A, 12B, and 12C for the purposes of this paper (see “Results” section for genomic coordinates). All but one of these was downregulated in males. Fifteen of the 24 were classified as estrogen-responsive in our analysis. Our data and an examination of the genomic distribution of these miRNA genes could further segregate the 12A and 12B clusters into a region particularly responsive to estrogen. Nine of the 16 miRNAs in these clusters were classified as estrogen-responsive in our analysis, while the sex-bias of an additional four miRNAs appeared to be chromosomal, together accounting for 76% of the miRNAs in these two clusters.

It is possible that the miRNAs within each of the three clusters are transcribed as a polycistronic primary miRNA transcript. It is even possible that all three clusters are transcribed as one very large transcript; the *Esr1* gene itself spans more than twice the distance as these clusters. If this were the case, Esr1 could regulate the expression of all of the clustered miRNAs through actions at a single promoter. However, examination of the location of TSSs across this locus argues against this. Beginning just 250-bp upstream of mir-673, there are 10 TSSs within cluster 12A alone [35]. Individual TSSs for mir-433 and mir-127 have also been identified in an independent study [57]. It seems unlikely that estrogen-induced suppression of these miRNAs occurs through actions at each of their promoters; it is more likely that the effects of estrogen are mediated by broader epigenetic changes to the local chromatin structure. Support for the susceptibility of these miRNAs to epigenetic regulation can be found in a study that demonstrated chromatin modifying drugs could activate mir-127 expression in multiple human cancer cell lines [58]. Whether the hypothesized epigenetic alterations to this locus could persist beyond the neonatal window is not known. But taken together, these data suggest that the entire locus is epigenetically regulated by estrogen.

### Transcriptome-wide mapping of Argonaute footprints by Ago HITS-CLIP

Identifying the gene transcripts targeted by an individual, or group, of miRNAs is the first step in understanding the biological processes they regulate and their functional relevance. Unfortunately, estimates of error in target prediction made by many popular algorithms can range from ~30 to 60% [20, 24]. At the same time, the importance of accuracy in this process was recently highlighted in a study by Bleazard et al. which identified biases in target prediction algorithms that led to the significant enrichment of certain functional categories of predicted gene targets of even randomly generated lists of miRNAs [59]. We avoided these biases in bioinformatics approaches by empirically characterizing the miRNA-mRNA interactome of the neonatal hypothalamus utilizing Ago HITS-CLIP. This technique involves UV irradiation to covalently crosslink RNA-protein complexes, followed by immunoprecipitation of Ago-RNA complexes, and subsequent high-throughput sequencing. This allows us to constrain potential participants in predicted miRNA-target connections to only those portions of the transcriptome bound by Ago and the subset of mature miRNAs loaded into the RISC complex [24].

We faced several challenges in adapting the Ago HITS-CLIP technique to our model system. First, in focussing on the neonatal hypothalamus, we were constrained by the fact that this brain region is both smaller and more heterogeneous than tissues used in previous HITS-CLIP experiments. In addition, unlike with many more commonly used assays, the analysis workflow for Ago HITS-CLIP experiments is not well established. To maximize the signal-to-noise ratio in our target prediction, we utilized two criteria to identify “robust” Ago footprints for downstream analyses. The first, footscore, was the average number of reads for each footprint across treatment groups. The second, biological complexity, was the number of treatment groups in which the footprint was present. Biological complexity, adapted from the original Ago HITS-CLIP study, was used to establish reproducibility for what was essentially a binary outcome; an Ago footprint was either present or absent in a treatment group [24]. A histogram of the average footscore for each Ago footprint revealed a wide range and an extremely long-tailed distribution, with a high proportion of footprints having low footscores. Importantly, we found that Ago footprints with high footscores were more likely to be identified in more than one group (biological complexity ≥ 2). Therefore, we established footscore and biological complexity thresholds that excluded all but the 20% most prevalent Ago footprints that were also present in at least two of our three experimental groups for downstream analyses. We identified 11,720 distinct robust Ago footprints that fit these criteria in 6689 transcripts. Forty-eight percent of these targeted mRNAs contained more than one footprint, with an average of 1.8 footprints/transcript, though 16% contained ≥ 3. It is likely that we have dismissed many legitimate footprints in the M/Veh group that were not present in the F/Veh and M/Form groups if miRNA-mediated silencing/degradation had proceeded to the extent that they were no longer detectable. In addition, the broad reduction in miRNA expression we found in males could account for a large number of Ago footprints unique to the M/Veh group. But these data are strikingly similar to the 11,463 footprints at a rate of 2.3 per transcript identified in mouse cortex by Chi et al. in the original Ago HITS-CLIP study and suggest that we are probably erring on the side of being more conservative in our selection of criteria for robust Ago footprints [24]. Finally, a qualitative comparison of Ago footprints across groups was consistent with the intermediate miRNA expression phenotype that resulted with aromatase inhibition, in that the M/Form group shared 59 and 63% of footprints with F/Veh and M/Veh, respectively, while F/Veh and M/Veh groups shared only 47%. The observed impact of estrogen on miRNA expression, in combination with a broader impact on gene transcription, likely accounts for this effect.

### Sex-biased miRNAs regulate genes relevant to sexual differentiation of the brain

We utilized two approaches to integrate our miRNA expression and Ago HITS-CLIP data to identify candidate miRNA-gene target connections functionally relevant to sexual differentiation of the neonatal hypothalamus. In the first, we started with genes known to mediate steroid signaling in the brain, and then determined if they contained Ago footprints that were predicted targets of sex-biased miRNAs. Not surprisingly, robust Ago footprints were present in transcripts encoding estrogen receptor alpha (*Esr1*), estrogen receptor beta (*Esr2*), androgen receptor (*Ar*), and aromatase (*Cyp19a1*), though not in the progesterone receptor (*Pgr*). Importantly, five of the six footprints in these mRNAs were located in the target’s 3′ UTR. Footprints in all four genes were predicted targets of sex-biased miRNAs. In fact, the only footprint that was not connected to a sex-biased miRNA, Esr2, begins only 13 nucleotides downstream of a second footprint, and it is probable that these are really a single functional miRNA recognition element.

Unfortunately, experimental validation of miRNA-target interactions lags far behind their prediction, though there are curated databases of validated connections. We queried DIANA-Tarbase v7.0 for validated connections to *Esr1*, *Esr2*, *Ar*, and *Cyp19a1* to compare with our results [41]. Validated connections were only available for *Esr1* and *Ar*. Eleven connections to *Esr1* have been validated across two experiments, and 13 connections to *Ar* in a single experiment [32, 60]. Both of these studies utilized Ago HITS-CLIP, though one was performed in the liver and the second in myoblasts. Underscoring the tissue-specific nature of gene expression, there was no overlap of validated connections to *Ar* between our study and the database, nor was there any overlap in *Esr1* connections between the two experiments compiled in the database. However, the connection we identified between *Esr1* and the sex-biased miR-206-3p was also found in myoblasts [60]. mir-206 also regulates Esr1 in human breast cancer [61, 62]. While we categorized it as chromosomally regulated, these studies also showed that Esr1 agonists suppress mir-206 expression, suggesting Esr1 and mir-206 are participants in a mutually inhibitory feedback loop. In this context, activation of mir-206 blocks estrogen-induced cell proliferation. This also appears to be the case in the neonatal hypothalamus, where the top four Ago HITS-CLIP-predicted targets of miR-206-3p are the proto-oncogene Bcl2, Bmp4, Rspo1, and Smarca4; all of which are involved in regulating cell death and proliferation. The sex-specific regulation of cell death plays a well-characterized role in sexual differentiation of at least two hypothalamic nuclei, the sexually dimorphic nucleus of the preoptic area and anteroventral periventricular nucleus [63–65]. mir-206 could play an important role in mediating the impact of estrogen on these processes.

In a second approach toward integrating our miRNA expression and HITS-CLIP data, we asked whether genes targeted by estrogen-responsive miRNAs converged on specific biological processes. We then used these data to identify a network of miRNA-target interactions with the potential to mediate the impact of estrogen on hypothalamic sexual differentiation. We operated on the assumption that the expression and subsequent activity of the genes targeted by estrogen-suppressed miRNAs would be activated and so focused on the targets of miRNAs that were downregulated by estrogen. Two clusters of biological processes predominated processes involved in macromolecule metabolism/gene expression and developmental processes. Specifically, 380 targets were annotated with the GO term Gene Expression, 321 with the term System Development, and a subset of 156 of these targets were annotated by both terms. Together, these annotations, with obvious relevance to hypothalamic sexual differentiation, accounted for 45% of the targets of estrogen-downregulated miRNAs.

Network analysis of the interactions between estrogen-downregulated miRNAs and their targets annotated with Gene Expression and/or System Development revealed that many genes were targets of more than one miRNA. This could reflect cooperative regulation by groups of miRNAs with shared functionality. Another equally interesting possibility is that these interactions are more exclusive at the level of specific regions and cell types within the neonatal hypothalamus. Two illustrative examples of genes targeted by multiple estrogen-responsive miRNAs are Tet2 and Zfp488. Tet2 is targeted by three estrogen-downregulated miRNAs (miR-500-3p, miR-1196-3p, and miR-3060-5p), and as a key component of cytosine demethylation pathways, it can have a widespread impact on epigenetic gene regulation [66]. For example, through its regulation of 5-hydroxymethyl-cytosine levels at specific genetic loci, Tet2 is required for the reprogramming of induced pluripotent stem cells [67]. ZFP488 is a transcription factor involved in oligodendrocyte differentiation and is targeted by four estrogen-downregulated miRNAs (miR-93-5p, miR-217-3p, miR-665-5p, and miR-3072-5p) [68]. Widespread sex differences in white matter content and neuronal connectivity have been found in human imaging studies [4, 69]. In addition, pubertal gonadal steroids, including estrogen, have been linked to sex differences in axon myelination [70, 71]. Estrogen regulation of oligodendrocyte differentiation in the neonatal hypothalamus, perhaps setting the stage for later sex-specific patterns of myelination, could be mediated by its actions on sex-biased miRNAs and the downstream targeting of transcription factors such as ZFP488.

## Conclusions

Sexual differentiation of the brain, and that of stress circuitry in the hypothalamus specifically, during the perinatal-sensitive period seems to be particularly susceptible to environmental programming effects [23] [46]. It has been hypothesized that disruption of the sex-specific masculinization of this circuitry resulting from insults, such as prenatal stress exposure, may drive observed sex differences in human neurodevelopmental disease [3, 72]. Estrogen is the primary driver of the sexual differentiation of the male brain during the perinatal-sensitive period. Surprisingly, this single hormone drives diverse programs of sex-specific development that vary widely across different cell types and across the sexually dimorphic male brain, which neuroscientists have only begun to scratch the surface of [11]. The fundamental complexity that must be at the source of this phenomenon suggests that additional layers of regulation are acting downstream of estrogen to mediate this incredible and unique specificity. In this study, we demonstrated that the neonatal hypothalamic miRNA environment is dynamically responsive to organizational estrogen. Using Ago HITS-CLIP to map connections between estrogen-responsive miRNAs and target genes at a transcriptome-wide level, we have uncovered novel candidate regulators of prototypical mediators of estrogen-driven sexual differentiation of the brain, including Esr1 and Cyp19a1. Integrating miRNAs and their broad actions on gene function into our conceptualization of the factors directing sexual differentiation of the brain could be a highly informative next step in efforts to understand the complexities behind these processes.

## Additional file

Additional file 1: Table S1. Ago HITS-CLIP identified connections between sex-biased miRNA and their target genes (robust Ago footprints). (CSV 542 kb)

## Abbreviations

Ago: Argonaute
*Ar*: Androgen receptor
BC: Biological complexity
*Cyp19a1*: Aromatase
*Esr1*: Estrogen receptor α
*Esr2*: Estrogen receptor β
F/Veh: Female/Vehicle
GO: Gene Ontology
HITS-CLIP: High-throughput sequencing of RNA isolated by cross-linking immunoprecipitation
M/Form: Male/formestane
M/Veh: Male/vehicle
miRNA: microRNA
OPLS-DA: Orthogonal partial least squares discriminant analysis
*Pgr*: Progesterone receptor
PN2: Postnatal day 2
RISC complex: RNA-induced silencing complex
RPM: Reads per million
TSS: Transcription start site
